# Protective Action of *Ostreococcus Tauri* and *Phaeodactylum Tricornutum* Extracts towards Benzo[a]Pyrene-Induced Cytotoxicity in Endothelial Cells

**DOI:** 10.3390/md18010003

**Published:** 2019-12-18

**Authors:** Manon Le Goff, Antoine Delbrut, Marie Quinton, Rémi Pradelles, Maelle Bescher, Agnès Burel, Benoît Schoefs, Odile Sergent, Dominique Lagadic-Gossmann, Eric Le Ferrec, Lionel Ulmann

**Affiliations:** 1 EA 2160 Mer Molécules Santé—MIMMA, IUML FR-3473 CNRS, Le Mans Université, F-53020 Laval, France; manon.le_goff@univ-lemans.fr (M.L.G.); 2 Univ Rennes, Inserm, EHESP, Irset (Institut de recherche en santé, environnement et travail)—UMR_S 1085, F-35000 Rennes, France; maelle.bescher@univ-rennes1.fr (M.B.); odile.sergent@univ-rennes1.fr (O.S.); dominique.lagadic@univ-rennes1.fr (D.L.-G.); 3 Microphyt, 713 Route de Mudaison, 34630 Baillargues, France; antoine.delbrut@microphyt.eu (A.D.); marie.quinton@microphyt.eu (M.Q.); remi.pradelles@microphyt.eu (R.P.); 4 Univ Rennes, Biosit–UMS 3480, US_S 018, F-35000 Rennes, France; agnes.burel@univ-rennes1.fr (A.B.); 5 EA 2160 Mer Molécules Santé—MIMMA, IUML FR-3473 CNRS, Le Mans Université, F-72000 Le Mans, France; benoit.schoefs@univ-lemans.fr (B.S.)

**Keywords:** *Ostreococcus tauri*, *Phaeodactylum tricornutum*, benzo[a]pyrene, endothelial cells, cell protection, cytokines, cell viability, mRNA expression, extracellular vesicles, aryl hydrocarbon receptor

## Abstract

Marine microalgae are known to be a source of bioactive molecules of interest to human health, such as *n*-3 polyunsaturated fatty acids (*n*-3 PUFAs) and carotenoids. The fact that some of these natural compounds are known to exhibit anti-inflammatory, antioxidant, anti-proliferative, and apoptosis-inducing effects, demonstrates their potential use in preventing cancers and cardiovascular diseases (CVDs). Benzo[a]pyrene (B[a]P), a polycyclic aromatic hydrocarbon (PAH), is an ubiquitous environmental pollutant known to contribute to the development or aggravation of human diseases, such as cancer, CVDs, and immune dysfunction. Most of these deleterious effects are related to the activation of the polycyclic aromatic hydrocarbon receptor (AhR). In this context, two ethanolic microalgal extracts with concentrations of 0.1 to 5 µg/mL are tested, *Ostreoccoccus tauri* (OT) and *Phaeodactylum tricornutum* (PT), in order to evaluate and compare their potential effects towards B[a]P-induced toxicity in endothelial HMEC-1 cells. Our results indicate that the OT extract can influence the toxicity of B[a]P. Indeed, apoptosis and the production of extracellular vesicles were decreased, likely through the reduction of the expression of CYP1A1, a B[a]P bioactivation enzyme. Furthermore, the B[a]P-induced expression of the inflammatory cytokines IL-8 and IL1-β was reduced. The PT extract only inhibited the expression of the B[a]P-induced cytokine IL-8 expression. The OT extract therefore seems to be a good candidate for counteracting the B[a]P toxicity.

## 1. Introduction

Polycyclic aromatic hydrocarbons (PAHs), generated during the incomplete combustion of organic matter, constitute an ubiquitous family of environmental contaminants. Humans are exposed to PAHs through various sources, including water and skin contact but mainly through ambient air and food [1]. PAHs are known to contribute to the development or aggravation of human diseases, such as cardiovascular diseases (CVDs), cancer, and immune dysfunction [2,3,4]. Benzo[a]pyrene (B[a]P), the prototype of polycyclic aromatic hydrocarbons, is classified into group 1, i.e., carcinogenic to humans, by the International Agency for Research on Cancer (IARC), and is among the priority toxic substances according to the US Environmental Protection Agency (US-EPA) as well as the World Health Organization (WHO) [5,6]. B[a]P exerts a wide range of toxic effects, including carcinogenic, pro-inflammatory, and apoptotic effects [7,8]. Most of these deleterious effects are related to the activation of the polycyclic aromatic hydrocarbon receptor (AhR) [9]. The binding and activation of AhR by B[a]P induces the transcription of many genes involved in its own metabolism, including enzymes that metabolize xenobiotics, such as cytochrome P450s (e.g. CYP1A1).

Numerous studies have shown that diet can modulate the response of organisms to the absorption, distribution, metabolism, and excretion of xenobiotics [10,11]. Furthermore, some diets containing pigments (carotenoids: astaxanthin, fucoxanthin, and so forth) and long-chain polyunsaturated fatty acids (eicosapentaenoic acid [EPA] and docosahexaenoic acid [DHA]), also present in microalgae, have shown their effectiveness in preventing CDVs and modulating carcinogenesis [12,13,14]. Moreover, numerous studies have suggested that many natural dietary compounds are able to prevent or reduce the adverse effects of xenobiotics on human health [15]. Indeed, it has been shown that polyunsaturated fatty acids (PUFAs) played a protective role in B[a]P-induced carcinogenesis by significantly reducing DNA adduct levels [16]. It has also been reported that the green alga *Chlorococcum humiloca*, rich in carotenoids, inhibits the genotoxicity of B[a]P in human lymphocytes [17]. Thus, these studies support the hypothesis that microalgae can be used as a protective agent. The prevention of food of a chemically related toxicity is an attractive strategy to counteract the development of diseases and promote health [18,19]. Although microalgae have desirable biological activities to control diseases like CVDs, information on the effectiveness of their protective action towards toxicity induced by environmental pollutants is still lacking.

Microalgae are photosynthetic microorganisms living mainly in aquatic environments. They are used for the production of biofuel, for the biomerization of polluted environments, in aquaculture, and cosmetology, but also as food supplements for animal and human nutrition [20,21,22,23]. Although the number of microalgal species is estimated between 200,000 and 800,000, only a few are used for food applications [21]. At present, in the European Union, the microalgae *Odontella aurita, Arthrospira sp.*, *Chlorella sp.*, and *Tetraselmis chuii* are authorized as food algae and the oils extracted from *Schizochytrium*, *Ulkenia,* and *Haematococcus pluvialis* have been approved as novel food ingredients [24]. Hence, there is a real major issue to study new microalgal species with a view to their potential exploitation as new human food products. Microalgal culture has also several advantages. Indeed, these microorganisms have a high growth rate, their cultivation does not use arable land, and they already are considered an interesting alternative source to fishery resources [25].

In addition, the composition of microalgae was revealed to be an excellent source of useful by-products such as PUFAs, pigments, antioxidants, lipids, and vitamins from their own metabolism [26]. Moreover, some of these metabolites have shown biological activities for human health, including antiviral, antimicrobial, antioxidant, anti-inflammatory, anticancer, and CVD prevention properties [26,27,28].

The green microalga *Ostreococcus tauri* (OT) belongs to the *Mamiellophyceae* that is the major class in the picoeukaryotic phytoplankton [29]. In this microalga, genes involved in the omega-3 PUFA synthesis, with front-end delta-6 and delta-4 desaturases [30] and elongases involved in C18 and C20 PUFAs conversion, have been identified and characterized [31]. According to these metabolic pathways, OT is able to synthesize 16:4*n*-3, 18:4*n*-3, and 22:6*n*-3 that are the most predominant fatty acids, representing more than 39% of total fatty acids with about 13% of 22:6*n*-3 [32]. This microalga is also rich in pigments such as chlorophyll b, Mg-2,4-divinyl pheoporphyrin, and unusual carotenoids, the composition of which being not firmly established as micromonal, neoxanthin, dihydrolutein, prasinoxanthin, and uriolide [33,34]. Among these, neoxanthin has been shown to reduce cell viability through apoptosis induction in human prostate cancer cells [35] and extracts containing prasinoxanthin were reported to have antioxidant properties [36].

*Phaeodactylum tricornutum* (PT) is a marine pennate diatom belonging to the class of the *Bacillariophyceae*. This microalga is used on an industrial scale in aquaculture feed because of its high content of PUFAs, and specifically EPA [37,38,39]. PT is also rich in carotenoids, mainly fucoxanthin, whose concentration is at least ten times higher than that of macroalgae compared to dry matter [40]. Many studies have shown that fucoxanthin has antioxidant, anti-tumor, anti-inflammatory, and anti-obesity effects [41]. Fucoxanthin extract from PT has been reported to have anti-inflammatory, antioxidant and anti-proliferative effects on blood mononuclear cells and on different cell lines [42]. The fact that OT and PT contain several metabolites known to have effects on human health, such as PUFAs and carotenoids, makes them of interest for disease prevention. Moreover, the presence of a mixture of these metabolites could have synergistic effects in regulating the parameters involved notably in CVDs or cancers [43].

Actually, no study has been reported on the use of extracts from the microalgae *O. tauri* and *P. tricornutum* in the prevention of CVDs induced by environmental pollutants. Thus, in this study, an endothelial cell model (HMEC-1) is used to study the potential chemopreventive effects of OT and PT extracts against adverse effects induced by B[a]P. We choose endothelial cells because they are present in multiple homeostatic functions, including the formation of blood vessels, the regulation of blood coagulation, platelet function, vascular tone, inflammatory reactions, and neoangiogenesis, in addition to their barrier function [44]. Endothelial dysfunction is associated with serious human pathologies, such as inflammation and atherosclerosis pathologies, which are themselves associated with exposure to environmental pollutants [4,45,46,47,48,49], and they are a well-known target of B[a]P.

The results of this study provide a better understanding of the chemopreventive effects of these two microalgal extracts on B[a]P-induced toxicity, thus supporting the use of new candidates for the prevention of diseases like CVDs induced by B[a]P exposure.

## 2. Results

### 2.1. Carotenoid and Fatty Acid Contents in P. tricornutum and O. tauri Extracts

Total chlorophylls and carotenoids contents in PT and OT ethanol extracts were determined by UV-Vis spectrophotometer (Table 1 and Table 2). The main pigment detected in the two microalgal extracts is chlorophyll *a* with 5.08 g/L for PT extract and 2.51 g/L for OT extract, respectively. The presence of other chlorophylls have been identified in the extracts, chlorophyll c at 0.47 g/L for PT extract and chlorophyll b at 1.64 g/L for OT extract. The total carotenoid content in the PT extract is twice that found in the OT extract. The carotenoid composition of both extracts agrees with the algal carotenoid composition reported in the literature [50,51]. They differ between the extracts both qualitatively and quantitively. Indeed, the PT extract contains a high concentration (2.13 g/L) of fucoxanthin, the main carotenoid in this microalga. The presence of β-carotene was also detected (at 0.03 g/L) in the PT extract. In the OT extract, β-carotene at a higher concentration (0.05 g/L) than in the PT extract was also detected. Other carotenoids were detected in the OT extract but not quantified because their molar extinction coefficients in acetone were not reported [50,52]. Thus dihydrolutein, micromonal, prasinoxanthin, and neochrome were detected. Neochrome is an artifact formed from neoxanthin that is present in the OT extract by the acid-catalyzed rearrangement during isolation [52].

The total fatty acids composition of *P. tricornutum* and *O. tauri* extracts were determined by gas chromatography (Table 3). As expected, the PT extract showed a higher content of C20:5*n*-3 (EPA) representing 40 mol % of total fatty acids in the extract. The second major fatty acid found in this extract was palmitoleic acid (C16:1*n*-7, PAL), hexadecatetraenoic acid (C16:4) and palmitic acid (C16:0) with 18, 11, and 7 mol % of total fatty acids, respectively. For the OT extract, the predominant fatty acid was oleic acid (C18:1, OA) with 23 mol % of total fatty acids. Unlike in the PT extract, the levels of stearidonic acid (C18:4*n*-3, SDA), stearic acid (C18:0), α-linolenic acid (C18:3*n*-3, ALA), and DHA (C22:6*n*-3) were of 11.5, 11, 8.5, and 7.7 mol %, respectively, in the OT extract.

### 2.2. Cytotoxic Effects of B[a]P and Microalgal Extracts on Endothelial HMEC-1 Cells

**Figure 1:**
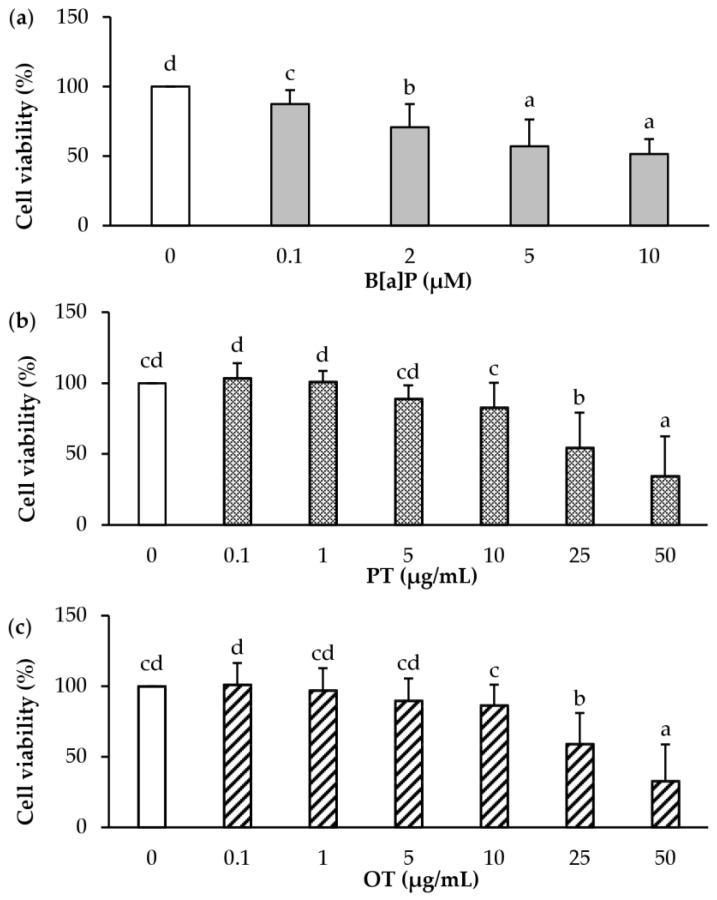
Cytotoxicity of B[a]P, *P. tricornutum*, and *O. tauri* extracts in endothelial HMEC-1 cells. HMEC-1 cells were exposed to vehicle (DMSO) or different concentrations of (**a**) B[a]P 0.1 µM to 10 µM or (**b**) *P. tricornutum* (PT) extract and (**c**) *O. tauri* (OT) extract at 0.1 to 50 µg/mL for 24 h. Results are represented as mean values ± SD from at least 3 independent experiments. Mean values assigned with different letters are significantly different (*p* < 0.05) with a < b < c < d.

The next set of experiments was carried out in order to determine the cytotoxic effects of B[a]P and PT and OT extracts on endothelial HMEC-1 cells. After treatment for 24 h with various concentrations of B[a]P (0.1, 2, 5, 10 μM) and microalgal extracts (0.1, 1, 5, 10, 25, 50 μg/mL), cell viability was measured by MTT assay in HMEC-1 cells. As shown in Figure 1a, B[a]P at 2 μM significantly decreased the cell viability by 29% (Figure 1a). For microalgal extracts alone, beyond 10 μg/mL of PT and OT extracts, a significant decrease in cell viability was observed (Figure 1b,c). Following this experiment, we chose, for the rest of the experiments, the concentration of 2µM B[a]P, which allows us to determine whether microalgal extracts influenced the toxicity induced by B[a]P. For microalgal extracts, concentrations at 0.1, 1 and 5 µg/mL that do not have a significant toxic effect, were chosen to study the potential protective action of the extracts.

### 2.3. Effect of Co-Exposure to B[a]P and Extracts of P. tricornutum or O. tauri on the Viability of Endothelial HMEC-1 Cells

**Figure 2:**
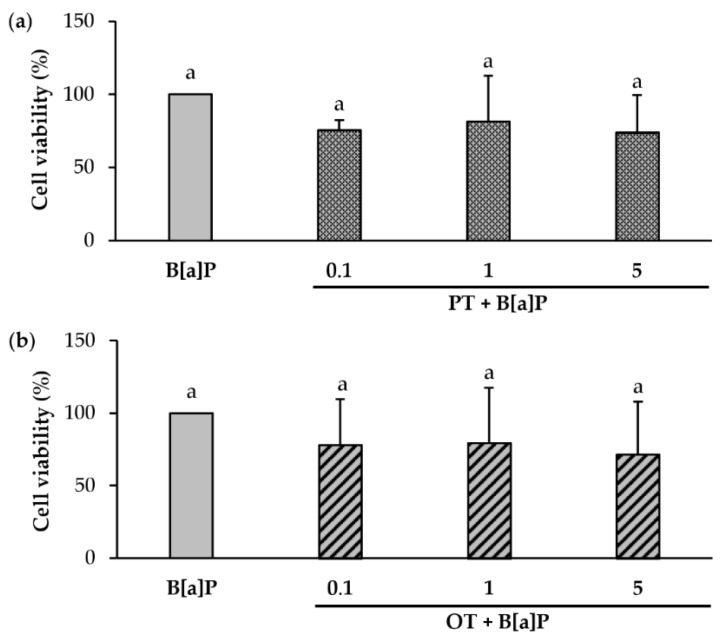
Effect of co-exposure to B[a]P and extracts of *P. tricornutum* or *O. tauri* on cytotoxicity in endothelial HMEC-1 cells. HMEC-1 cells were exposed to 2 µM B[a]P and (**a**) *P. tricornutum* (PT) extract and (**b**) *O. tauri* (OT) extract at 0.1 to 5 µg/mL for 24 h. Cell viability upon B[a]P treatment was set at 100% and then compared with HMEC-1 cells co-exposed with microalgal extracts. Results are represented as mean values ± SD from at least 3 independent experiments. Mean values assigned with the same letters are not significantly different (*p* < 0.05).

To verify whether microalgal extracts could influence the cytotoxicity of B[a]P, HMEC-1 cell viability after co-exposure to 2 µM B[a]P and each extract (0.1, 1 and 5 µg/mL) for 24 h was compared to that of cells exposed to 2 µM B[a]P alone. PT and OT extracts, at any tested concentration, did not significantly influence the cytotoxicity of B[a]P (Figure 2a,b).

### 2.4. Effect of P. tricornutum or O. tauri Extracts on Gene Expression of Pro-Inflammatory Cytokines Induced by B[a]P

**Figure 3:**
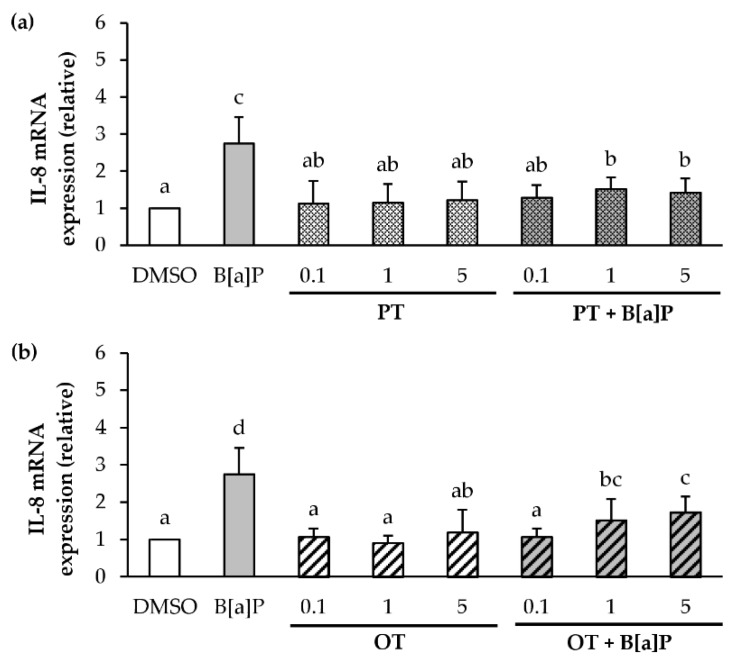
Effects of *P. tricornutum* and *O. tauri* extracts on B[a]P-induced IL-8 mRNA expression. mRNA expression of IL-8 was analyzed using RT-qPCR. Data are expressed relatively to mRNA levels of IL-8 found in corresponding control cells (DMSO), arbitrarily set to 1 unit. HMEC-1 cells were exposed to vehicle (DMSO) or treated with 2 μM B[a]P, or with 0.1, 1 and 5 µg/mL *P. tricornutum* (PT) extract (**a**) or *O. tauri* (OT) extract (**b**), or with a combination of the toxicant and the extract for 24 h. Results are represented as mean values ± SD from at least 3 independent experiments. Mean values assigned with different letters are significantly different (*p* < 0.05) with a < b < c < d.

**Figure 4:**
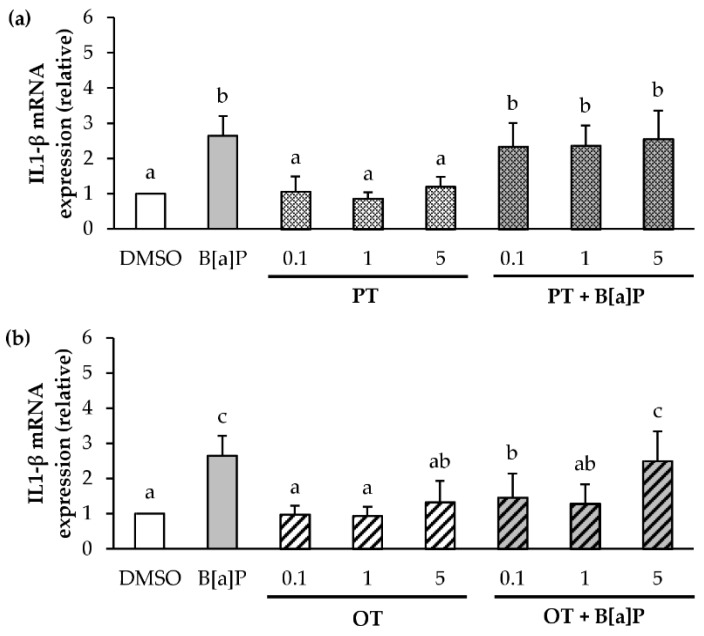
Effects of *P. tricornutum* and *O. tauri* extracts on mRNA expression of IL1-β in endothelial HMEC-1 cells. mRNA expression of IL1-β was analyzed using RT-qPCR. Data are expressed relatively to mRNA levels of IL1-β found in corresponding control cells (DMSO), arbitrarily set to 1 unit. HMEC-1 cells were exposed to vehicle (DMSO) or treated with 2 μM B[a]P, or 0.1, 1 and 5 µg/mL *P. tricornutum* (PT) extract (**a**) or *O. tauri* (OT) extract (**b**), or with a combination of the toxicant and the extract for 24 h. Results are represented as mean values ± SD from at least 3 independent experiments. Mean values assigned with different letters are significantly different (*p* < 0.05) with a < b < c.

It has been reported that B[a]P is able to induce the expression of pro-inflammatory cytokines, notably in endothelial cells [53,54,55,56], and that some extracts of microalgae may have anti-inflammatory properties [57,58]. It was therefore decided to study the effects of B[a]P at 2 μM and microalgal extracts on the gene expression of several pro-inflammatory cytokines (TNF-α, IL-6, IL-8 and IL-1β) and cyclooxygenase COX-2. In endothelial HMEC-1 cells, 2 µM B[a]P did not induce COX-2, TNF-alpha, and IL-6 mRNA expressions (data not shown), unlike IL-8 and IL-1β (Figure 3 and Figure 4).

Microalgal extracts (0.1, 1 and 5 µg/mL) were evaluated for their ability to affect the increased IL-8 and IL-1β mRNA transcription induced by 2 µM B[a]P. PT extract alone in a range of 0.1 to 5 μg/mL (Figure 3a) did not induce the expression of IL-8 mRNA compared to the control (DMSO). With regard to co-exposure with B[a]P, the PT extract significantly inhibited B[a]P-induced IL-8 mRNA expression whatever the concentration tested. Concerning the OT extract alone (Figure 3b), no induction of IL-8 mRNA expression was found. As for the PT, in combination with B[a]P, the OT extract decreased the B[a]P-induced IL-8 mRNA expression. 

Regarding IL-1β mRNA expression, both microalgal extracts (0.1, 1 and 5 μg/mL) did not induce IL-1β mRNA expression compared with the control (Figure 4a,b). However, in the presence of co-exposure, the PT extract (Figure 4a) did not influence the mRNA expression of IL-1β induced by B[a]P, whatever the concentration tested. In contrast, the OT extract at 0.1 and 1 μg/mL decreased this expression (Figure 4b). However, the OT extract at 5 µg/mL did not affect the increased IL1-β mRNA transcription induced by 2 µM B[a]P. These results showed that the microalgal extracts could inhibit the gene expression of several pro-inflammatory cytokines induced by B[a]P in endothelial cells.

### 2.5. Only the O. tauri Extract Inhibits the B[a]P-Induced Apoptosis

**Figure 5:**
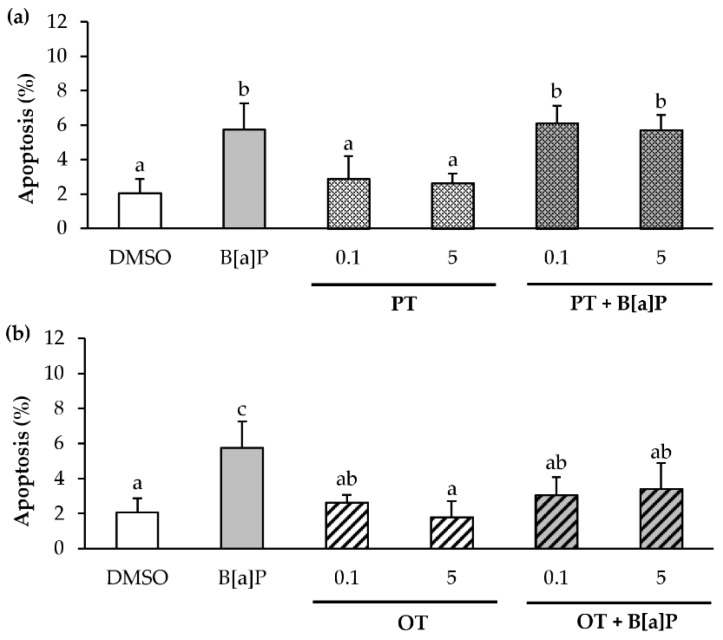
Effects of *P. tricornutum* and *O. tauri* extracts on B[a]P-induced apoptosis in endothelial HMEC-1 cells. HMEC-1 cells were exposed to vehicle (DMSO) or treated with 2 μM B[a]P, or with 0.1, 1 and 5 µg/mL *P. tricornutum* (PT) extract (**a**) or *O. tauri* (OT) extract (**b**), or with a combination of the toxicant and the extract for 24 h. Apoptotic nuclei were analyzed by fluorescence microscopy after staining with Hoechst 33342. Results are represented as mean values ± SD from at least 3 independent experiments. Mean values assigned with different letters are significantly different (*p* < 0.05) with a < b < c.

Since B[a]P is known to induce apoptosis [59,60], and it is also known that microalgal extracts can inhibit apoptosis in cancerous cells [61], we therefore decided to evaluate apoptosis after exposure for 24 h to B[a]P or microalgal extracts (0.1 and 5 μg/mL) alone or in co-exposure. This was performed by using fluorescence microscopy after Hoechst staining (Figure 5). Consistent with the literature, 2 μM B[a]P slightly increased the percentage of apoptotic cells by 3-fold compared with control. In contrast, the PT extract alone did not affect the apoptosis level (Figure 5a). Interestingly, the PT extract had no effect on B[a]P-induced apoptosis whatever the extract concentration used. Concerning the OT extract (Figure 5b), no induction of apoptosis was observed, whatever the concentration used. However, an inhibition of B[a]P-induced apoptosis with the OT extract at 0.1 and 5 µg/mL was observed. These results suggested that the OT extract, unlike the PT extract, could inhibit B[a]P-induced apoptosis in HMEC-1 cells.

### 2.6. Effect of P. tricornutum or O. tauri Extracts on the Induction of CYP1A1 by B[a]P

**Figure 6:**
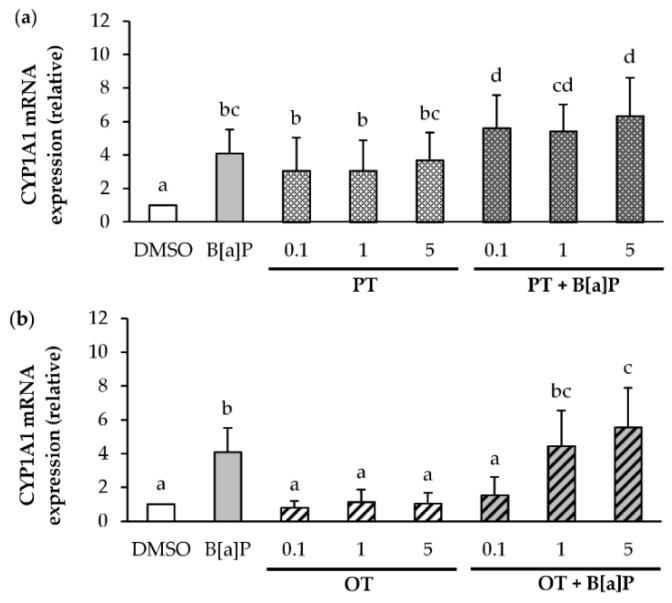
Effects of *P. tricornutum* and *O. tauri* extracts on B[a]P-induced CYP1A1 mRNA expression. mRNA expression of CYP1A1 was analyzed using RT-qPCR. Data are expressed relatively to mRNA levels of CYP1A1 found in corresponding control cells (DMSO), arbitrarily set to 1 unit. HMEC-1 cells were exposed to vehicle (DMSO) or treated with 2 μM B[a]P, or with 0.1, 1 and 5 µg/mL *P. tricornutum* (PT) extract (**a**) or *O. tauri* (OT) extract (**b**), or with a combination of the toxicant and the extract for 24 h. Results are represented as mean values ± SD from at least 3 independent experiments. Mean values assigned with different letters are significantly different (*p* < 0.05) with a < b < c < d.

Since B[a]P is metabolized by cytochrome P450 enzymes, such as CYP1A1, resulting in the formation of several metabolites, and is partly responsible for its apoptotic effects [8] and its carcinogenicity [62,63]. The effects of B[a]P or microalgal extracts (0.1, 1 and 5 μg/mL) alone or in co-exposure were investigated on CYP1A1 mRNA expression in endothelial cells exposed during 24 h (Figure 6). As expected, B[a]P at 2 μM induced the expression of CYP1A1 in endothelial HMEC-1 cells compared to the DMSO control (Figure 6). As shown in Figure 6a, the levels of CYP1A1 mRNA were markedly increased in endothelial cells treated with PT extract alone or in co-exposure when compared with their DMSO counterpart. In addition, co-exposure is more inductive than exposure to the PT extract alone. For the OT extract alone (Figure 6b), no change in CYP1A1 mRNA level compared to DMSO control was observed. However, in co-exposure, the OT extract at 0.1 μg/mL inhibited the induction of B[a]P-induced CYP1A1 mRNA. This effect was no longer observed with the highest concentrations of extracts (1 and 5 μg/mL). Thus, the OT extract at a concentration of 0.1 µg/mL would appear to be able to inhibit the expression of CYP1A1 to mRNA unlike the PT extract.

### 2.7. O. tauri Extract Inhibits the Release of Extracellular Vesicles

**Figure 7:**
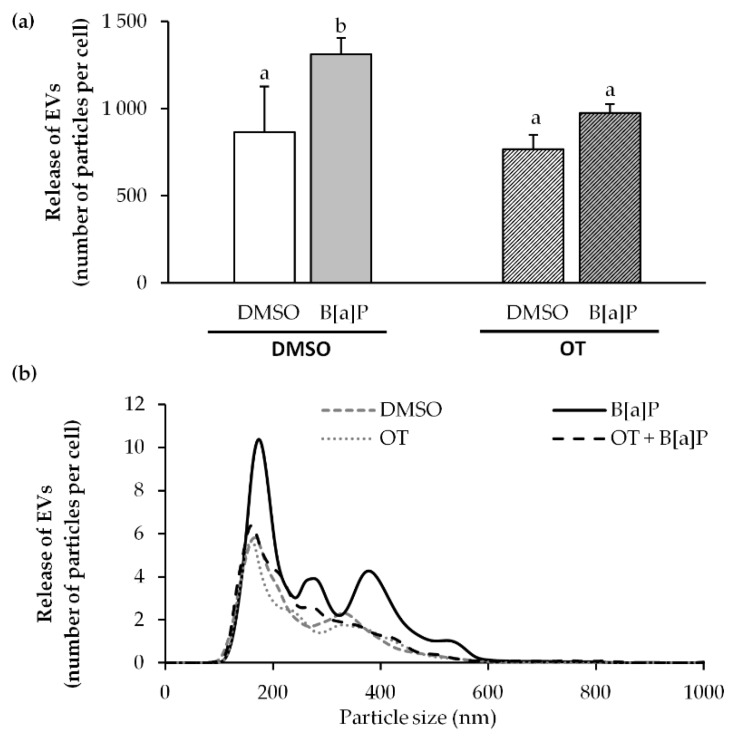
Effects of B[a]P and *O. tauri* extracts on the EVs released by endothelial HMEC-1 cells. HMEC-1 cells were exposed to vehicle (DMSO), 2 µM of B[a]P, 0.1 µM/mL of *O. tauri* (OT) extracts or with a combination of the toxicant and the extract for 24 h. Total EVs were isolated by ultracentrifugation and analyzed by Nanoparticle Tracking Analysis (NTA). (**a**) EV production released per HMEC-1 cells exposed with B[a]P or/and OT. (**b**) Representative size distribution profile by NTA of EVs produced by endothelial cells. Results are represented as mean values ± SD from at least 3 independent experiments. Mean values assigned with different letters are significantly different (*p* < 0.05) with a < b.

According to the above results, such as IL-8, IL-1B, CYP1A1 mRNA expression and apoptosis, the 0.1 μg/mL OT extract seems to be more prone to regulate the adverse effects from B[a]P. Therefore, our work finally focused on the effect of this extract at 0.1 µg/mL to study the production of extracellular vesicles (EVs). It is known that B[a]P is responsible for endothelial dysfunction [54,64]. In addition, it has been observed that endothelial dysfunction can cause an increased shedding of endothelial extracellular vesicles that are markers of the pathophysiological state of the producing cells [65,66]. Thus the effects of B[a]P and the OT extract on EV production by the endothelial cells were investigated following a 24 h treatment. As expected from our previous work [67], NTA revealed that 2 μM B[a]P significantly stimulated EV production, unlike the OT extract used at 0.1 μg/mL (Figure 7a). In the co-exposure, an inhibition of the production of EVs induced by B[a]P was observed. NTA observation of EVs size distribution pattern (Figure 7b) showed that they had a diameter ranging from 100 nm to 600 nm, and that the OT inhibited the production of the entire EV population induced by the B[a]P regardless of their size.

## 3. Discussion

Food supplementation is a promising strategy to use natural protective agents that can offer health benefits. Among these promising agents, microalgae are becoming increasingly important. Indeed, the growing interest in the use of microalgae in human health comes from their composition that is rich in molecules of interest (lipids, polysaccharides, carotenoids) known to have beneficial effects on health [27]. In addition, many studies reveal the pharmacological interest of microalgae as natural products that can offer significant benefits in CVDs, inflammation, and cancer [68,69,70,71,72]. In this context, our study aimed at evaluating the capacity of two microalgal extracts from the green alga *Ostreococcus tauri* (OT) and the diatom *Phaeodactylum tricornutum* (PT), to prevent harmful effects induced by an environmental pollutant, B[a]P, known to contribute to the development or aggravation of human pathologies notably CVDs [2,3]. To do so, we used a human endothelial cell model.

The two microalgal compositions tested differ in their pigment, particularly carotenoids, and fatty acid compositions. The PT extract was shown to be rich in fucoxanthin and EPA, while the OT extract is found to be rich in neoxanthin and ALA, SDA, OA, and DHA, all molecules of interest for human health [33,34,37,38,40,73].

To assess the protective effects of the OT and PT extracts on B[a]P-induced toxicity, extracts at non-toxic concentrations had to be used. When cells were exposed to the extracts alone, a cytotoxic effect was observed only at high concentrations (25–50 µg/mL). Previous in vitro studies have shown that *n*-3 PUFAs at micromolar concentrations are toxic to several cell lines [74,75]. Moreover, it has also been reported that stearic acid and palmitic acid induces cytotoxicity in human aortic endothelial cells (HAEC) [76]. Since our extracts were rich in n-3 PUFAs, DHA for OT, and EPA for PT, but also in saturated fatty acids, and even if the fatty acid concentrations were not calculated, it might be hypothesized that the cytotoxicity observed at the highest concentrations could be attributed to these fatty acids present in both microalgal extracts.

We also verified that co-exposure to B[a]P and either extract did not induce further loss of viability as estimated by MTT test (a measure of mitochondrial activity; cf. Materials and Methods), thus validating our experimental conditions.

Since B[a]P is known to induce immune dysfunctions and microalgal extracts are involved in anti-inflammatory activities, their influence was next studied on the expression of several pro-inflammatory cytokines such as IL-8 and IL-1β. Both extracts inhibited the mRNA expression of B[a]P-induced IL-8 in HMEC-1 cells. Moreover, only the OT extract inhibited IL-1β expression induced by B[a]P. Under our test conditions, these results suggest that the OT and PT extracts have a potential to suppress inflammation by inhibiting the expression of pro-inflammatory cytokines. Despite the presence of several studies showing that microalgal extracts are anti-inflammatory, their extremely diversified composition, particularly in pigments and fatty acids, makes it difficult to attribute the biological effects observed to a single bioactive molecule contained in the extracts. However, pigments present in our extracts, such as chlorophyll α, β-carotene, and fucoxanthin have already shown anti-inflammatory activity [77,78,79,80]. Furthermore, previous studies have shown that PUFAs, such as DHA, ALA, and PAL can inhibit the expression of IL-6, IL-8, and IL-1β in many cellular models [81,82,83,84]. Since OT and PT extracts contain these pigments and these fatty acids, it is likely that there are synergistic effects between these bioactive molecules that can contribute to anti-inflammatory effects. It is interesting to note that many studies have linked the anti-inflammatory effects of compounds contained in microalgae with the inhibition of the NF-κB signaling pathway. A study on human bronchial epithelial cells also showed that EPA, by inhibiting MAPKs/NF-κB signaling, inhibits IL-8 induced by cigarette smoke extracts [85]. In another study, fucoxanthin was reported to reduce pro-inflammatory mediator levels via the inhibition of NF-κB pathway activation in murine macrophages [69]. Knowing that B[a]P is known to activate the NF-κB pathway via the production of reactive oxygen species (ROS), leading to the induction of pro-inflammatory cytokines such as IL-8 and IL-1β [54,86], it is also likely that the anti-inflammatory effects observed with our microalgal extracts might be due to an inhibition of the NF-κB pathway via the presence of PUFAs and carotenoids in our extracts.

Cytochromes P450 and in particular CYP1A1 are of crucial importance for the metabolic activation and detoxification of many xenobiotics, such as PAHs, including through the ARNT pathway (AHR/aryl hydrocarbon nuclear translocator). Modulating the expression of this bioactivation enzyme may be an appropriate strategy to prevent toxic effects and the development of CVDs, inflammation, and cancer [87,88]. In this context, we investigated the influence of microalgal extracts on the metabolism of B[a]P and, in particular, the expression of CYP1A1. Only the PT extract alone induced CYP1A1 mRNA expression but did not influence B[a]P-induced CYP1A1 induction. The induction of CYP1A1 expression could be related to the presence of carotenoids and in particular the presence of high fucoxanthin in the PT extract. Indeed, in previous studies, Satomi et al. [89], showed that fucoxanthin significantly induced CYP1A1 mRNA in the hepatocellular carcinoma cell line HepG2. Concerning the OT extract, an inhibition of the B[a]P-induced CYP1A1 mRNA expression was observed in our endothelial cell model, at a concentration of 0.1 µg/mL only. It has previously been demonstrated that co-treatment of DHA and B[a]P resulted in an increase in CYP1A1 and CYP1B1 mRNA expression and B[a]P metabolism, resulting in increased DNA adduct levels in rat liver epithelial F258 cells [90]. However, it was also found in HUVEC cells, another endothelial cell model, that supplementation with DHA significantly decreased the B[a]P-induced CYP1A1 mRNA expression [91]. The fact that studies show that co-exposure to DHA and B[a]P could exert opposite effects on B[a]P-induced CYP1A1 mRNA expression thus could be cell-type dependent.

Moreover, an inhibitory effect has been observed only at the lowest concentration of OT. Molecules other than lipids and pigments are present in microalgae and in particular flavonoids [92]. These molecules can act as AhR agonists and effectively induce AhR activation and CYP1A1 production [93]. Although the flavonoid composition has not been investigated in our microalgal extracts, it can thus be hypothesized that since CYP1A1 is regulated by AhR activation, the flavonoids present in the OT extract, at higher concentrations, might become competitors for AhR activation and CYP1A1 induction with regard to B[a]P.

It has already been shown that B[a]P induces death in many cell models [94,95]. We therefore decided to study the effect of microalgal extracts on B[a]P-induced apoptosis in our endothelial model. B[a]P induced apoptosis in HMEC-1 cells unlike cells exposed to microalgal extracts alone. We have shown that only the OT extract protected against B[a]P-induced apoptosis. Interestingly, it is well known that the activation of AhR and CYP1A1 plays an important role in B[a]P-induced apoptosis [95]. Moreover, the fact that only the OT extract and not the PT one was able to inhibit CYP1A1 expression is consistent with the inhibition of B[a]P-induced apoptosis. Thus, the inhibition of CYP1A1 expression and apoptosis induced by the OT extract may be due to its high DHA content, as previously demonstrated [16]. In addition, the OT extract contains other fatty acids, such as ALA and OA, not present or weakly present in the PT extract. These fatty acids have already shown protective effects against apoptosis. Indeed, ALA offered a protective effect of H_2_O_2_-induced apoptosis by inhibiting the NF-κB signaling pathway in HAECS endothelial cells [96]. OA also showed an inhibitory effect of stearic acid-induced apoptosis in these same cells [97]. Thus, the presence of these fatty acids in the OT extract may contribute with DHA to the anti-apoptotic effect observed with the OT extract.

Several studies have shown that damage to endothelial cells can lead to an increase in the release of extracellular vesicles (EVs) that are markers of the pathophysiological state of the producing cells [98]. Moreover, we have recently demonstrated that B[a]P is able to stimulate the production of EVs from endothelial cells [67] and to increase membrane fluidity by cholesterol-depletion involved in the release of EVs in B[a]P-treated hepatocytes [99]. The fact that the OT extract seems more inclined to regulate the harmful effects of B[a]P, the production of EVs under the influence of this extract was then studied. We found that it inhibited such a production. The influence of *n*-3 PUFAs on the production of EVs has already been reported. Indeed, it has previously been described by Wu et al. [100] that fish oil supplementation (rich in EPA and DHA) reduces the number of circulating endothelial extracellular vesicles. The inhibition of EVs by the OT extract could be due to the presence of high levels of *n*-3 PUFAs, among which is DHA, as has been reported with fish oil supplementation [79]. These results suggest that the OT extract is able to reduce endothelial damage induced by B[a]P. Interestingly, it has previously been demonstrated that DHA can interfere with B[a]P-induced membrane remodeling [90]. Since membrane remodeling is a key element in EV production, it is possible that the DHA present in our OT extract inhibited the B[a]P-induced membrane remodeling and hence the related release of EVs.

Overall, our data indicate that the OT extract can reduce the toxicity of B[a]P by decreasing the expression of the inflammatory cytokine (IL-8, IL1-β), apoptosis, the B[a]P bioactivation enzyme CYP1A1, and extracellular vesicle production. The PT extract inhibited only the B[a]P-increased expression of the pro-inflammatory cytokine IL-8. According to our data, the OT extract seems to be a good candidate as a dietary component that can reduce B[a]P-induced toxicity, making it of interest for the prevention of diseases, such as CVDs, induced by exposure to this environmental pollutant. However, further research is needed to determine whether the observed effects are mainly due to fatty acids (e.g. PUFAs *n*-3) or to carotenoids present in OT extract, or whether it is due to a synergistic effect of these molecules. Although the objective of this study was not to conduct an in vivo study, this would be necessary to complete this work. Indeed, an in vivo study would allow us to get closer to human nutritional protocols. In addition, these protocols would allow us to evaluate the bioavailability and bio accessibility of pigments and PUFAs provided by our microalgal extracts. These two steps are important factors to know and understand the real biological role of these molecules.

## 4. Materials and Methods

### 4.1. Chemicals and Reagents

Benzo[a]pyrene (B[a]P), dimethyl sulfoxide (DMSO) were obtained from Sigma-Aldrich (Saint-Quentin Fallavier, France), and MTT (Thiazolyl Blue tetrazolium bromide) was provided from Interchim (Montluçon, Fance). All other chemicals used in this study were purchased from commercial sources at the highest purity available. Chemicals were prepared as stock solutions in DMSO. The final concentration of vehicle did not exceed 0.2% (*v*/*v*); control cultures received the same concentration of DMSO.

### 4.2. Microalgal Cultivation

The marine microalgae PT and OT were grown by Microphyt (Baillargues, France) in 5000 L photobioreactors (PBR) consisting of 1.2 km of glass tubes with co-circulation of liquid medium and CO_2_ enriched air [101,102]. The PBR was set under a greenhouse in order to control the temperature (between 20 °C and 30 °C) and the intensity of the natural light by using curtains. The pH set point of 7.5 was automatically controlled by CO_2_ injection and monitored by an inline Fermprobe F-235 pH probe from Broadley James (Silsoe, United Kingdom). Air was injected continuously at a rate of 20 L·min^−1^. The culture medium used is a marine type medium, corresponding to a modified *f*/2 medium. Cells were harvested by bowl centrifugation at 6,000 rpm at room temperature (maximum reachable temperature of 35 °C) using a KG 8006 centrifuge from GEA (Oelde, Germany) and concentrated at the rate of around 15%–20% dry weight. The biomass was frozen at −20 °C in polyethylene bags, and stored at −20 °C before freeze-drying.

### 4.3. Preparation of Ethanolic Extracts

The extractions were carried out in a dark room, at a temperature of 25 °C, under nitrogen and with agitation for 24 h. The solvent used is absolute ethanol at a ratio of 10:1 (*v*:*w*). The suspension was then centrifuged at 4000 rpm for 5 min, and the supernatant was filtered through a membrane with a porosity of 0.22 µm. The filtrate was recovered and the ethanol evaporated under nitrogen away from light at 35 °C. This crude extract was diluted in absolute ethanol to obtain a final crude extract concentration of 55.9 and 75.5 mg/mL for the extract of PT and OT, respectively. The solutions thus obtained were stored at 4 °C in the dark after inserting the head space with nitrogen. For the treatment of endothelial cells, the ethanol contained in the microalgal extracts was evaporated using a Concentrator plus from Eppendorf. After sonication on the ice, 50 mg of ethanolic extract was evaporated and centrifuged during 10 min. The extract was collected in DMSO in order to obtain an extract concentration of 50 mg/mL. The extracts were closed under argon and stored at −20 °C protected from light.

### 4.4. Fatty Acid Composition Analyses

Total fatty acid compositions were analyzed by gas chromatography coupled with a flame ionization detection (GC-FID). After the evaporation of the solvent from the microalgal extracts, 1 mL of 0.5 mol/L NaOH was added to the samples. The lipids were saponified by incubating the samples for 20 min at 80 °C under nitrogen. The transesterification process was carried out using 2 mL of 14% boron trifluoride in methanol (MeOH-BF_3_) at 80 °C for 20 min under nitrogen. Then 1 mL of isooctane and 1 mL of 35% NaCl were added and, after shaking, the samples were centrifuged. The upper phase was carefully transferred to the anhydrous sodium sulphate column for drying samples. Fatty acid methyl esters (FAMEs) were analyzed by using a FOCUS gas chromatography instrument (Thermo Electron Corporation, Les Ulis, France) equipped with a capillary column CP Sil-88 25 m × 0.25 mm (Varian, Les Ulis, France) and a flame-ionization detector (FID). Chromatograms were analyzed using the Azur version 4.6.0.0 software package (DATALYS, France). Each fatty acid was identified from an authentic fatty methyl ester standard (Sigma-Aldrich, Saint-Quentin Fallavier, France) and results were expressed as a molar percentage (mol%).

### 4.5. Pigment Composition

The total pigment amount was determined spectroscopically using a double beam Lambda 25 UV/VIS spectrometer (PerkinElmer). The concentration of chlorophyll (Chl) a, Chl c and total carotenoids in the PT were calculated according to Heydarizadeh et al. [103], whereas the Chl a, Chl b and total carotenoids in the OT were calculated according to Lichtenthaler et al. [104].

The separation of the pigments present in the microalgal extracts was carried out by thin-layer chromatography (TLC). Five microliters of OT and PT extracts were applied 1 cm from the base of a pre-coated TLC-plate silica G-25 (Macherey-Nagel, Germany, 20 cm × 20 cm) and allowed to dry for a few minutes in the dark. Then, the plates were developed in a closed glass chamber in the dark using acetone/petroleum ether (30%/70%) (*v*/*v*) as the mobile phase [105]. Individual bands were scrapped from the TLC plate, eluted into 1 mL acetone and then centrifuged (10,000× *g* for 10 min at 4 °C). The absorption spectra of the pigments extracted from individual bands were determined by spectrophotometry between 400–800 nm using a double beam Lambda 25 UV-Vis spectrometer (PerkinElmer, Villebon-sur-Yvette, France). The reference was acetone. The positions of the absorbance maxima were determined after the calculation of the second derivative absorbance spectrum according to Schoefs and Franck [106]. Pigment identification was based on the comparison of the absorption maxima found in this study and in the literature (see Table 1 and Table 2) [52,107].

### 4.6. Cell Culture

The human Microvascular Endothelial Cell line (HMEC-1) was obtained from the Center for Disease Control and Prevention (Atlanta, GA, USA). Cells were maintained in MCDB-131 medium containing 10% fetal bovine serum (FBS), L-glutamine (10 mM), hydrocortisone (1 µg/mL), penicillin (50 unit/mL), streptomycin (50 unit/mL), and epidermal growth factor (10 ng/mL). At 90% confluence, cells were cultured overnight in serum-free medium before treatment the day after.

### 4.7. Cell Viability Assay

The cell viability was performed by MTT assay. MTT cell-viability assay measures the reduction of a tetrazolium component into an insoluble formazan product by the mitochondria of viable cells. Cells were incubated with different concentrations of microalgal extracts (0.1 to 50 µg/mL), B[a]P (0.1 to 10 µM) alone or in co-exposure with B[a]P at 2 µM and microalgal extracts at 0.1 to 5 for 24 h. Cells were rinsed with PBS and incubated for 2 h at 37 °C with an MTT solution (0.5 mg/mL in a serum-free medium). After washing, cells were lysed with DMSO. The absorbance was measured by using the SPECTROstar Nano BMG LABTECH at 560 nm with a reference wavelength of 670 nm.

### 4.8. RNA Isolation and Analysis

Total RNA was isolated from HMEC-1 cells using TRIzol method (Invitrogen, Cergy-Pontoise, France). RNA samples (1 µg) was reverse transcribed into cDNA using the High-Capacity cDNA Reverse Transcription Kit (Life Technologies, Carlsbad, CA, USA). Quantitative PCR (qPCR) assays were next performed using SYBR Green PCR Master Mix on the CFX384 Touch^TM^ Real-Time PCR Detection System (Bio-Rad, Hercules, CA, USA). The mRNA expressions were normalized by means of 18 s mRNA levels. The 2^-∆∆ct^ method was used to express the relative expression of each selected gene. Sequences of the tested primers are reported in Table 4.

### 4.9. Apoptosis

Apoptotic death in HMEC-1 cells was assessed by the visualization of chromatin condensation after nuclear staining. Cells used for apoptosis analysis were cultured in 6-well plates. After exposure to B[a]P and/or microalgal extracts, the cells were stained with 50 μg/mL Hoechst 33,342 in the dark at 37 °C for 30 min; cells were then examined by fluorescence microscopy (ZEISS Axio Scope A1 microscope). Over 300 cells in randomly selected microscopic fields were analyzed per condition of treatment.

### 4.10. Isolation of Extracellular Vesicles

Cells were cultured in 151.9 cm^2^ cell culture Petri dishes (Corning ™, Thermo Fisher Scientific, France). After treatment with 2 μM B[a]P and/or 0.1 µg/mL OT extract for 24 h, culture supernatants were recovered to isolate the EVs. Cell debris present in conditioned medium was removed by centrifugation at 3650× *g* for 10 min. EVs were pelleted by direct ultracentrifugation of the culture medium at 100,000× *g* for 1 h 45 min at 4 °C (Beckman Coulter Optima L-90K Series Ultracentifuges, rotor Sw 28.1). The EV pellet was then resuspended and washed with sterile phosphate-buffered saline (PBS). The EVs were then centrifuged at 100,000× *g* for 1 h 45 min at 4 °C and the EV pellet was recovered in sterile PBS.

### 4.11. Nanoparticle Tracking Analysis

The number and size distribution of EVs were performed using Nanoparticle Tracking Analysis (NTA). EV samples were diluted in sterile PBS before analysis with NanoSight LM10 HS (Malvern Instruments, Malvern, UK). All samples were measured in triplicate at 25 °C and used the same instrument settings. Three videos of 30 s per sample were analyzed with NanoSight NTA 3.1 software (Malvern, UK) setting the detection threshold at 3. The results presented are the average of the three videos and three independent EV samples. Concentrations and distributions of EVs were then normalized to cell counts and expressed as numbers of EVs released per cell.

### 4.12. Electron Microscopy

Transmission electron microscopy (TEM) was performed as described by Théry et al., 2006 [108]. In summary, formvar carbon-coated copper grids (Agar Scientific, Stansted, UK) were placed for 20 min on a 5 μL EVs suspension previously resuspended in 2% paraformaldehyde (PFA). After washing with PBS, EVs were fixed in 1% glutaraldehyde in PBS for 5 min. After washing with distilled water, grids were placed on a drop of uranyl oxalate for 5 min, then on a drop of 2% methylcellulose/4% uranyl acetate (1/9; *v*/*v*) on ice for 10 min. EVs were visualized using a JEOL JEM-1400 transmission electron microscope (JEOL, Japan).

### 4.13. Characterization of Extracellular Vesicles

**Figure 8:**
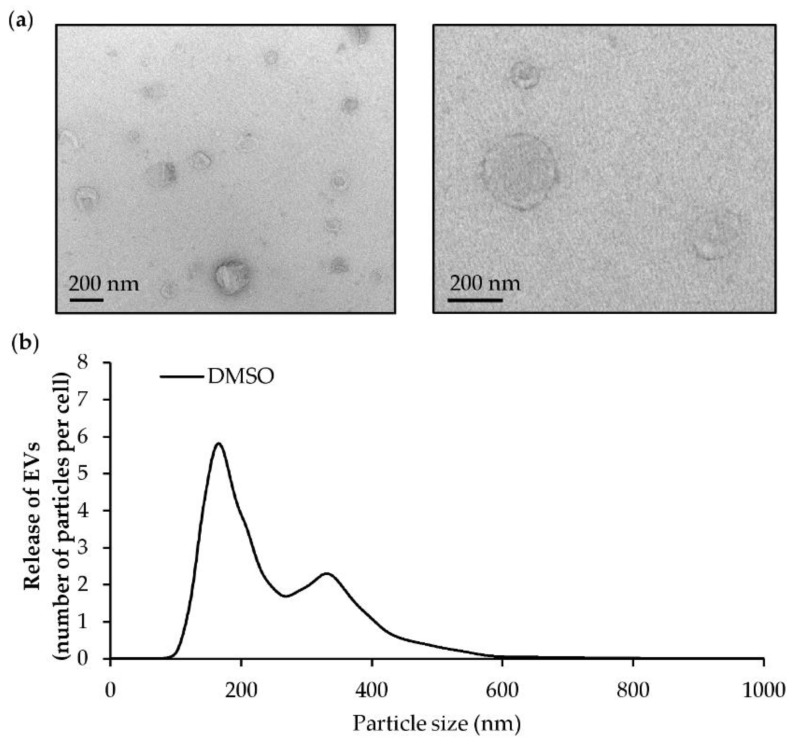
Characterization of extracellular vesicles (EVs) released by endothelial HMEC-1 cells. HMEC-1 cells were exposed to DMSO for 24 h. EVs were isolated by differential ultracentrifugation. (**a**) Transmission electron microscopy pictures of EV pellets (scale bars = 200 nm). (**b**) Representative size distribution profile by nanoparticle tracking analysis (NTA) of EVs produced by endothelial cells.

The culture supernatant was collected after 24 h of treatment with the vehicle (DMSO), and the EVs produced by HMEC-1 were isolated. In line with current knowledge in the field of EVs [109], two approaches have been used to confirm the presence and isolation of EVs in samples. After isolation, transmission electron microscopy (TEM) analysis confirmed the presence of EVs in the culture supernatant (Figure 8a). The characteristic morphology of EVs, cup-shaped, was observed. In addition to morphology, the number and size distribution of EVs was determined by NTA (Figure 8b). The population of EVs has been found to range from 100 nm to 600 nm with an average size of 257 ± 16 nm. The size and morphology characteristics of EVs suggested that endothelial cells HMEC-1 were able to produce EVs and that the developed method for isolated EVs was effective.

### 4.14. Statistical Analysis

All values were presented as means ± standard deviation (SD) from at least three independent experiments. After the analysis of variance by one-way ANOVA, the mean values were compared using Fisher’s least significant difference post hoc test (LSD). All statistical analyses were performed using Statgraphics Plus 5.1 (Manugistics Inc., Rockville, MD, USA).

## Figures and Tables

**Table 1:** Chlorophyll contents *of P. tricornutum* and *O. tauri* extracts. Results are represented for an independent experiment. * Concentrations are calculated according to the literature.

Chlorophylls	Pigment Concentration (g/L)	
	PT	OT
Chlorophyll a	5.08	2.51
Chorophyll b *	-	1.64
Chorophyll c *	0.47	-

**Table 2:** Carotenoid contents *of P. tricornutum* and *O. tauri* extracts. Results are represented for an independent experiment.

Carotenoids	Absorbance Maxima (nm)	Absorbance Maxima in Literature (nm)	Pigment Concentration (g/L)	
			PT	OT
Total carotenoids			2.47	1.00
β-carotene	424,454,484	(429),454,480	0.03	0.05
Fucoxanthin	(415),443,472	(420),447,468	2.13	-
Other carotenoids				
Dihydrolutein	(−),432,462	408,428,453	-	0.08
Micromonal	422,451,477	(423),449,(472)	-	0.43
Prasinoxanthin	420,449,(−)	(426),459,(473)	-	0.10
Neochrome	(−),414,442	398,421,448	-	0.10

**Table 3:** Fatty acid composition of *P. tricornutum* and *O. tauri*. Results are represented as mean values ± SD for at least 3 independent experiments. ND: not detected.

Fatty Acids (mol %)	*P. tricornutum*		*O. tauri*	
	Mean	SD	Mean	SD
C14:0	1.18	0.43	4.20	2.80
C16:0	7.68	0.20	14.64	0.53
C16:1*n*-7	18.27	0.96	8.68	0.91
C16:2	5.28	0.29	ND	-
C16:4	11.23	1.38	ND	-
C18:0	1.88	0.32	10.91	2.69
C18:1	1.87	0.35	23.06	3.01
C18:2*n*-6	2.97	0.11	4.40	0.43
C18:3*n*-3	ND	-	8.56	0.86
C18:4*n*-3	ND	-	11.56	0.82
C20:1	ND	-	0.83	0.07
C20:2	2.08	0.26	ND	-
C20:4	3.34	0.16	0.97	0.26
C20:5*n*-3	39.91	2.91	1.16	0.18
C22:6*n*-3	1.72	0.11	7.69	1.26

**Table 4:** Primer sequences for RT-qPCR.

Gene Symbol	Forward Sequence	Reverse Sequence
CYP1A1	CCCACAGCACAACAAGAGACA	CATCAGGGGTGAGAAACCGT
IL-8	ACTCCAAACCTTTCCACCCC	TCTCAGCCCTCTTCAAAAACTTC
IL1-β	CTCTGGGATTCTCTTCAGCCA	AGGAGCACTTCATCTGTTTAGGG
18S	CGCCGCTAGAGGTGAAATTC	TTGGCAAATGCTTTCGCTC
